# Predictors of Stress, Anxiety, Depression and Quality of Life in Patients Diagnosed with Chronic Inflammatory Bowel Disease in Romania: A Cross-Section Observational Case-Report Study

**DOI:** 10.3390/jcm15051996

**Published:** 2026-03-05

**Authors:** Oliviu Florentiu Sarb, Adriana Daniela Sarb, Daniel Leucuta, Ciprian Brisc, Alina Ioana Tanțău

**Affiliations:** 1 Neuroscience Department, “Iuliu Hatieganu” University of Medicine and Pharmacy, 400012 Cluj-Napoca, Romania; sarboliviu@yahoo.com; 2 Department of Internal Medicine, 4th Medical Clinic, “Iuliu Hatieganu” University of Medicine and Pharmacy, 400012 Cluj-Napoca, Romania; 3 Department of Internal Medicine, Heart Institute, “Iuliu Hatieganu” University of Medicine and Pharmacy, 400001 Cluj-Napoca, Romania; adriana_porca@yahoo.com; 4 Medical Informatics and Biostatistics Department, “Iuliu Hațieganu” University of Medicine and Pharmacy, 400012 Cluj-Napoca, Romania; dleucuta@umfcluj.ro; 5 Gastroenterology Department, University of Medicine and Pharmacy, 410073 Oradea, Romania; brisciprian@gmail.com

**Keywords:** anxiety, depression, quality of life, chronic inflammatory bowel disease, ulcerative colitis, Crohn’s disease

## Abstract

**Background/Objectives:** Inflammatory bowel disease (IBD), mainly encompassing Crohn’s disease (CD) and ulcerative colitis (UC), is strongly linked to psychological comorbidities such as depression, anxiety, and stress. These mental health factors negatively impact disease progression, healthcare utilization, and quality of life (QoL). **Methods:** Participants completed the Depression–Anxiety–Stress Scale 21 (DASS-21) and EuroQol (EQ-5D-5L) questionnaires. Statistical analyses included multivariate linear regressions to identify predictors of psychological distress. **Results:** We conducted a cross-sectional case–control study involving 355 participants: 55 with CD, 90 with UC, and 210 healthy controls. Patients with IBD reported significantly higher levels of stress (*p* < 0.01), anxiety (*p* = 0.016), and depression (*p* < 0.01) compared to controls. Severe or very severe symptoms were more prevalent in those with CD and UC. The relative risk for stress was high (RR = 2.1), and the risk for depression was significantly elevated (RR = 1.54) in the IBD population. Quality-of-life analysis revealed lower EQ visual analog scale scores and increased difficulties across all domains, particularly in emotional well-being and pain. Multivariate analysis showed UC diagnosis, female sex, and corticosteroid use as predictors of higher stress and depression scores, while self-reported rest was consistently protective. **Conclusions:** This study confirms the psychological burden of IBD and underscores the importance of regular screening for stress, anxiety, and depression in clinical care. Self-reported rest emerged as a key protective factor, suggesting potential benefits from interventions targeting sleep quality and emotional support. Future research should explore longitudinal outcomes and personalized psychological interventions in IBD populations.

## 1. Introduction

Inflammatory bowel diseases (IBDs), primarily represented by Crohn’s disease (CD) and ulcerative colitis (UC), are significantly associated with depression, suggesting a bidirectional relationship between these conditions. Studies indicate a prevalence of 15.2% for depressive disorders and 21.6% for depressive symptoms among patients with IBD [1]. Genetically predicted depression increases the risk of developing IBD [2]. Conversely, they also have an elevated risk of depression [3]. Female gender, a lack of social support, and active disease are independent predictors of depression in patients with IBD [4]. Depression rates are higher during relapses compared to remission [1,5]. Depression adversely affects disease outcomes, increasing the risk of relapses, medical costs, and emergency department visits [4,5]. Immunosuppressive therapies, particularly anti-TNF treatments, can reduce depressive symptoms [6]. 

Anxiety is also significantly associated with IBD, with studies reporting a prevalence of 20.9%, which is higher than the 15% seen in the general population [7]. A bidirectional relationship exists between anxiety and IBD, with active disease strongly correlated with higher anxiety rates compared to remission [8]. Female gender, smoking, extraintestinal manifestations, and a history of surgical interventions independently predict anxiety in patients with IBD [9]. Anti-TNF and immunomodulator therapies have shown significant reductions in anxiety symptoms over periods of up to five years [10]. 

Stress significantly influences the onset and progression of IBD. Extended stressful events are linked to higher relapse rates (90% vs. 40% in low-stress individuals) [11]. Recent research highlights elevated stress rates among patients with IBD [11,12]. A survey on patients during the COVID-19 pandemic found that 63.7% reported increased stress, with 21.1% experiencing worsened gastrointestinal symptoms [13]. Early-life stressors are more common among patients with IBD, suggesting that childhood stress may contribute to disease development [14].

The mechanism linking IBD to psychological disorders includes gut–brain axis (GBA) disruption where intestinal inflammation and brain function continuously influence each other via neural, immune, endocrine, metabolic, and microbiota pathways. Inflammation in the gut activates the enteric nervous system and the vagus nerve, altering emotional regulation and reducing vagal anti-inflammatory control, while circulating cytokines (TNF-α, IL-1β, and IL-6) can cross or signal through the blood–brain barrier, producing neuroinflammation and disrupting serotonin and dopamine signaling. Psychological stress further worsens disease activity by activating the HPA axis and cortisol release, increasing intestinal permeability and systemic inflammation. At the metabolic level, inflammation diverts tryptophan away from serotonin synthesis toward the kynurenine pathway, generating neurotoxic metabolites linked to depressive symptoms and fatigue. Finally, gut dysbiosis reduces beneficial short-chain fatty acids and alters production of neuroactive compounds, further modulating mood, anxiety, and stress and illustrating a self-reinforcing cycle between intestinal disease and mental health [15].

Given their high prevalence and impact in IBD, routine screening and management of psychological conditions are recommended as part of comprehensive patient care [8].

## 2. Materials and Methods

### 2.1. Study Design and Participants

To assess the impact of IBD on mental health, we conducted a cross-sectional case–control study (November 2023–February 2025) in Cluj-Napoca, Romania, involving both clinical university hospitals (Clinical County Emergency Hospital, Regional Institute of Gastroenterology and Hepatology “O. Fodor”) and a private medical center (DiGenio Institute). Medical staff distributed anonymous questionnaires to patients with confirmed IBD diagnoses during routine follow-up visits. The IBD group included adults (≥18 years) with confirmed diagnoses of CD or UC, based on the hospital registries (part of the national program), who consented to participate. The control group consisted of adults who presented in day-care services for minor gastroenterological complaints, had no diagnosis of IBD based on the hospital registries, and provided signed informed consent.

### 2.2. Data Collection and Instruments

All participants first completed a questionnaire collecting basic demographic information (e.g., age and sex), while patients with IBD also filled in a disease-specific section describing clinical characteristics. Psychological symptoms were evaluated using the Depression–Anxiety–Stress Scale (DASS-21), a self-report instrument composed of 21 statements divided into three subscales: depression, anxiety, and stress (7 items each) [16,17]. Participants rated how much each statement applied to them during the previous week on a 4-point Likert scale that ranged from 0 (“did not apply to me at all”) to 3 (“applied to me very much or most of the time”). Scores for each subscale were summed and multiplied by two to obtain the final severity scores, which were then interpreted according to established cut-offs (normal, mild, moderate, severe, and extremely severe).

### 2.3. Psychological and Quality of Life Assessment

Health-related quality of life was assessed using the EQ-5D-5L questionnaire. This instrument evaluates five dimensions—mobility, self-care, usual activities, pain/discomfort, and anxiety/depression—each rated on five levels ranging from no problems to extreme problems [18,19]. The combination of responses generates a five-digit health profile that can be converted into a single index value using population-based value sets, where higher values indicate better health status. Additionally, participants rated their overall perceived health on the EQ visual analog scale (EQ-VAS), which ranged from 0 (worst imaginable health) to 100 (best imaginable health).

### 2.4. Sample Size and Power Calculation

The sample size calculation was revised. Power analysis was performed a priori using G*Power version 3.1 for a three-group comparison on the primary outcome (depression), assuming α = 0.05, power = 80%, and a moderate effect size (f = 0.25), yielding a required minimum sample of 159 participants. The final sample (n = 355) exceeded this threshold, ensuring adequate power to detect small-to-moderate effects. 

### 2.5. Ethical Approval

This study was conducted in accordance with the principles of the Declaration of Helsinki and was approved by the University Ethics Committee (approval no. 52/28 February 2022). All participants provided informed consent prior to inclusion, and confidentiality and data protection regulations were respected throughout the research process. 

### 2.6. Statistical Analysis

Data were analyzed using IBM SPSS Statistics version 26. Continuous variables were first assessed for distribution normality (histograms, Q–Q plots, and normality tests). Normally distributed variables were summarized as the mean ± standard deviation, while non-normally distributed variables were expressed as the median and interquartile range (IQR). Categorical variables were reported as absolute counts and percentages.

Power analysis was performed for the primary outcome (depression) using a three-group comparison design (Crohn’s disease, ulcerative colitis, and controls) with a two-sided alpha of 0.05. With n = 55 (CD), n = 90 (UC), and n = 210 (controls), this study had >80% statistical power to detect small-to-moderate effect sizes (Cohen’s f ≈ 0.20; equivalent to Cohen’s d ≈ 0.40 in pairwise contrasts). This indicates that the sample size was adequate to identify clinically meaningful differences in depression scores across the three groups.

For comparisons between two independent groups, the independent sample *t*-test was used for normally distributed continuous variables, whereas non-parametric variables were analyzed using the Mann–Whitney U test. Comparisons between three groups (Crohn’s disease, ulcerative colitis, and controls) were performed using one-way ANOVA for normally distributed data and the Kruskal–Wallis test for non-normal distributions, followed by appropriate non-parametric post hoc pairwise comparisons with correction for multiple testing. Associations between categorical variables were evaluated using the Chi-square test or Fisher’s exact test when expected cell counts were small. The statistical significance threshold was set at *p* < 0.05.

To evaluate the relationship between disease group and psychological outcomes (depression, anxiety, and stress scores), multivariate linear regression models were constructed. When outcome residuals were not normally distributed, a square-root transformation was applied to improve model assumptions. Covariates included in the adjusted models were selected based on clinical relevance and the previous literature.

Model assumptions were systematically verified: multicollinearity was assessed using correlation matrices and variance inflation factors (VIFs); homoscedasticity was evaluated using the Breusch–Pagan test and inspection of residual plots; normality of residuals was examined with Q–Q plots; and linearity was checked using component-plus-residual plots.

## 3. Results

### 3.1. Demographic Characteristics of Groups

This study included 355 participants that were stratified into three groups, 55 with CD, 90 with UC and 210 in the control group. There were no significant differences in gender, age, residence environment, professional activity, body mass index (BMI) and sleep hours between groups. Demographic data is shown in Table 1.

### 3.2. Characteristics of the IBD Groups

Table 2 summarizes key clinical features of patients with CD and UC. Most patients did not follow a specific diet. Complications, such as fistulas, abscesses, and extraintestinal manifestations (uveitis, arthralgia, and sacroiliitis), were reported more frequently in the CD group.

### 3.3. Risk Factors Associated with CD, UC, and the Control Group

Risk factor analysis revealed some statistically significant differences between groups. The weight category was similar across groups. Smoking status showed no significant difference. Reported standard of living differed significantly, with patients diagnosed with CD and UC more likely to report poor or average living conditions, while the control group reported better living standards (Table 3).

### 3.4. DASS-21 Score Distribution and Risk of Psychological Disorders

DASS-21 analysis showed worse psychological symptoms in patients with IBD (Table 4).

Three separate analyses were conducted to assess the relative risk (RR) of depression, stress, and anxiety in patients with CD and UC versus controls.

◦ Depression was associated with a significantly increased risk (RR = 1.54, 95% CI: 1.17–2.02, *p* < 0.01), indicating a 54% higher likelihood of depression in patients with IBD. Both the CD (RR = 1.47, 95% CI: 1.02–2.13, *p* = 0.03) and UC (RR = 1.58, 95% CI: 1.16–2.14, *p* < 0.01) groups showed significantly higher risks compared to controls.
◦ Stress was associated with an even more pronounced risk (RR = 2.1, 95% CI: 1.54–2.87, *p* < 0.01), suggesting that patients with IBD are over twice as likely to experience severe stress. This pattern was consistent for both CD vs. the control (RR = 1.99, *p* < 0.01) and UC vs. the control (RR = 2.18, *p* < 0.01).
◦ Anxiety: The overall increased risk did not reach statistical significance (RR = 1.17, 95% CI: 0.97–1.42, *p* = 0.1). Patients with CD (RR = 1.11, *p* = 0.42) showed no significant difference, while patients with UC had a borderline increase (RR = 1.21, 95% CI: 0.97–1.49, *p* = 0.07) that was not statistically significant.

### 3.5. Quality-of-Life (QOL) Analysis

QOL scores (Table 5) revealed significant differences between the CD, UC, and control groups, highlighting the impact of IBD on general well-being. Overall health, assessed via the EQ-VAS, was significantly lower in patients with CD and UC compared to controls, with no difference between the CD and UC groups. Anxiety/depression symptoms were absent in 49.1% of patients with CD and 54.4% of patients with UC, compared to 81.4% in controls (Table 5). Usual activities were unaffected in 56.4% (CD), 66.7% (UC), and 84.3% (controls), with significant differences between IBD groups and controls. Self-care was less impacted but still showed significant group differences: 74.5% (CD), 77.8% (UC), and 89.5% (controls) reported no difficulties. Pain/discomfort was reported by 47.8% (CD), 52.2% (UC), and 21.4% (controls). Mobility was impaired in 21.8% (CD), 17.8% (UC), and 9.5% (controls), with significant differences overall, though UC vs. the control was not statistically significant.

### 3.6. Multivariate Analysis of Stress, Anxiety, and Depression Scores

To confirm the robustness of univariate results, multiple linear regression models were developed to predict depression, anxiety, and stress scores based on disease group, adjusted for demographic, behavioral, and biological predictors. The Durbin–Watson statistic ranged from 1.7 to 1.8 across models, indicating no significant autocorrelation. All variance inflation factors were near one, suggesting no multicollinearity.

In the multivariate model, stress scores were independently associated with disease group and selected covariates. Both UC (B = 1.040, *p* < 0.01) and CD (B = 0.725, *p* = 0.015) remained significant predictors compared with controls. Female sex (B = 0.718, *p* < 0.01) and younger age (B = −0.016, *p* < 0.01) were also significantly associated with higher stress scores. Corticosteroid treatment was positively associated with stress (B = 0.438, *p* = 0.023), while self-reported rest was negatively associated (B = −0.509, *p* < 0.01). Standard of living showed a positive association with stress (B = 0.253, *p* = 0.013). Smoking showed a borderline association (*p* = 0.06). Physical activity, alcohol consumption, BMI, relationship status, education level, urban residence, special diet, biologic therapy, and reported hours of sleep were not significantly associated with stress in the adjusted model. The model explained 21.0% of the variance in stress scores (R^2^ = 0.210; adjusted R^2^ = 0.160, *p* < 0.01) (Table 6).

Regarding anxiety levels, UC remained a significant predictor compared with controls (B = 0.686, *p* < 0.01), while CD did not reach statistical significance (*p* = 0.09). Female sex (B = 0.527, *p* < 0.01) and smoking (B = 0.509, *p* < 0.01) were significantly associated with higher anxiety scores. Self-reported rest was negatively associated with anxiety (B = −0.629, *p* < 0.01). Corticosteroid treatment, alcohol consumption, special diet, reported hours of sleep, physical activity, relationship status, standard of living, age, BMI, education level, urban residence, and biologic treatment were not significantly associated with anxiety in the adjusted model. The model explained 14.6% of the variance in anxiety scores (R^2^ = 0.146; adjusted R^2^ = 0.103, *p* < 0.01) (Table 7).

Regarding depression levels, UC remained a significant predictor compared with controls (B = 0.812, *p* < 0.01), while CD was not significantly associated (*p* = 0.36). Female sex (B = 0.642, *p* < 0.01) was positively associated with higher depression scores, whereas age was inversely associated (B = −0.016, *p* = 0.021). Self-reported rest was negatively associated with depression (B = −0.817, *p* < 0.01). Corticosteroid treatment, biologic therapy, physical activity, smoking, reported hours of sleep, relationship status, urban residence, standard of living, BMI, alcohol consumption, education level, and special diet were not significantly associated with depression in the adjusted model. The model explained 17.3% of the variance in depression scores (R^2^ = 0.173; adjusted R^2^ = 0.132, *p* < 0.02) (Table 8).

## 4. Discussion

This study successfully met its goal of collecting over 100 questionnaires per group (IBD vs. non-IBD). However, all groups showed suboptimal questionnaire completion, possibly reflecting limited participant engagement, perhaps due to the lack of incentives. Incomplete questionnaires were removed from the final analysis. This study included a relatively higher proportion of controls mainly to fulfill the conditions for multivariate analysis, which was the scope of this study, and to evaluate the predictors of stress, depression and anxiety. Patients with CD were lower in number than the patients with UC, but this might be explained by the lower incidence of the disease in Romania [20]. 

An interesting observation was that over half of the patients with UC reported alcohol consumption, with significant differences even compared to patients with CD. This could influence mental health, warranting more detailed investigation into future studies. Complications (intestinal and extra-intestinal) were more frequent in patients with CD, reflecting greater disease severity. Anxiety, depression, and stress scores from DASS-21 were significantly higher in both IBD groups compared to controls but not between CD and UC, aligning with the prior literature and this study’s aims [11,12,13,14,21,22].

DASS-21 findings confirmed a high prevalence of psychological symptoms in IBD, consistent with existing evidence. Stress scores showed a notably higher proportion of severe and very severe cases in the IBD groups. This aligns with findings from Black et al. [21], who reported stress as a trigger for symptom exacerbation and lower quality of life in patients with UC.

Regarding anxiety scores, while normal levels were comparable between groups, very severe anxiety was significantly more prevalent in patients with IBD. Moreover, stressful external situations such as the COVID-19 pandemic were associated with a significant decline in mental health, and high levels of anxiety in individuals with IBD underscore the importance of healthcare strategies during global crises [23].

Regarding the RR of developing a psychological disorder, our results demonstrate a significant increase in depression and stress among patients with IBD, while anxiety showed only a non-significant tendency toward higher values. The approximately 1.5-fold increased risk of depression observed in our cohort is consistent with large population-based and meta-analytic data reporting a similar magnitude of association between IBD and depressive disorders [1]. The stronger association found for stress supports previous evidence indicating that psychological distress represents a major complication of chronic intestinal inflammation and may exceed depressive symptoms in some patients, particularly during periods of disease activity [24]. In contrast, anxiety did not reach statistical significance, which aligns with the prior literature showing heterogeneous and often state-dependent anxiety risks in IBD, varying according to disease activity and patient characteristics rather than diagnosis alone [25]. Overall, our findings reproduce the commonly described gradient of psychological involvement in IBD, with depression consistently elevated, stress markedly increased, and anxiety more variable across populations [26].

Our findings highlight the psychological burden of IBD, supporting the need for early psychological interventions to improve disease management and quality of life

Quality-of-life analysis showed poorer general health in patients with IBD, which is unsurprising given the disease’s clinical manifestations and contributing factors like marital status and rest quality. Patients with IBD more frequently reported emotional issues, reduced ability to perform daily activities, impaired self-care and mobility, and more frequent pain/discomfort.

Multivariate analysis aimed to identify predictors of stress, depression, and anxiety. Self-reported rest was a protective factor across all outcomes. Biologic treatment was linked to lower stress and anxiety scores but had an inverse relationship with depression, potentially due to hidden medication effects or masking. Paradoxically, a better standard of living was associated with higher stress and anxiety, though not significantly. A slightly increased depression risk was also observed. This might relate to higher responsibilities or expectations associated with affluence. Alcohol consumption was linked to lower stress and anxiety but higher depression scores, though it was not statistically significant. Smoking emerged as a risk factor for all psychological outcomes, consistent with the literature noting its adverse effects on IBD progression [27,28].

Paradoxically, more reported sleep hours were associated with higher psychological scores. However, perceived rest was a strong protective factor, underscoring sleep quality over quantity, in line with previous studies [29].

One of the limitations of this study included difficult recruitment due to reluctance and incomplete questionnaires, limiting full engagement. A questionnaire for the sleep index was not present mainly because it would have decreased the number of questionnaires completed due to long completion times. In the first phase of the study, the first 30 participants completed a broader sleep questionnaire, but the data was not added to the final analysis as the research team decided to omit it due to poor completion ratios. Another limitation of this study was perhaps the lack of correlation between the clinical activity index and the stress, depression and anxiety scores. The absence of an IBD-specific quality-of-life questionnaire was due to the need to allow direct comparison with the control group, as disease-specific instruments cannot be applied to individuals without IBD. A final limitation was the lack of disease activity data, preventing stratification of patients with IBD into active vs. remission phases.

The high prevalence of depressive symptoms and stress identified in our cohort supports the need for systematic psychological screening in routine IBD care. Psychological comorbidity is frequently underrecognized in gastroenterology practice, despite its established association with disease activity, poorer quality of life, reduced treatment adherence, and increased healthcare utilization. Our findings reinforce that mental health evaluation should not be limited to patients reporting overt emotional distress; it should be incorporated as a standard component of disease monitoring.

Screening can be efficiently implemented using short validated instruments that require minimal time and training. Tools such as DASS-21, HADS, PHQ-9, or GAD-7 can be administered during outpatient visits or electronically prior to consultation. A practical approach would be periodic screening at diagnosis, during flares, after therapy escalation, and annually during remission. Patients exceeding predefined thresholds should undergo further evaluation rather than immediate psychiatric labeling, as psychological symptoms in IBD may reflect inflammatory activity, treatment effects, illness perception, or functional impairment [21]. Therefore, screening should function as a triage step guiding clinical attention rather than a standalone diagnostic procedure [30].

Following identification of psychological burden, management should adopt a multidisciplinary model. First-line measures include patient education, reassurance, and discussion of disease expectations, which alone may reduce illness-related stress. Structured psychoeducation improves coping strategies and reduces catastrophizing behaviors commonly observed in chronic diseases. When symptoms persist, referral for targeted psychological therapy is recommended. Cognitive behavioral therapy, gut-directed psychotherapy, and stress management programs have demonstrated beneficial effects on quality of life and emotional symptoms in IBD patients. Mindfulness-based interventions may also improve perceived disease control and reduce stress reactivity [21].

Pharmacologic therapy should be individualized and considered particularly in moderate-to-severe depressive symptoms or functional impairment. Selective serotonin reuptake inhibitors are most frequently used due to the tolerability and potential anti-inflammatory properties suggested in experimental models [30]. Importantly, treatment decisions should be coordinated between gastroenterologists, primary care physicians, and mental health specialists to avoid fragmentation of care. Integration of psychological management into IBD clinics has been shown to improve patient satisfaction and may reduce unnecessary investigations driven by symptom amplification.

Sleep quality and fatigue represent additional modifiable contributors to psychological burden and disease perception. Addressing sleep hygiene, circadian rhythm disturbances, and pain control may indirectly improve emotional well-being. Lifestyle factors including physical activity and dietary counseling should also be incorporated, as they influence both inflammatory activity and mental health through GBA mechanisms [31].

Future research should focus on longitudinal designs to clarify the temporal relationship between inflammation and psychological symptoms, distinguishing reactive emotional responses from inflammation-driven neurobehavioral changes. Interventional studies evaluating structured psychological programs integrated into IBD clinics are needed to determine whether early treatment alters disease course, healthcare utilization, and treatment adherence. Trials targeting modifiable factors such as sleep quality, fatigue, and stress resilience may provide clinically actionable strategies beyond pharmacologic therapy. Finally, biomarker-guided approaches combining inflammatory markers with psychological assessment may help identify patients most likely to benefit from early psychosocial intervention.

## 5. Conclusions

This study provides valuable insights into the prevalence of stress, depression, and anxiety in IBD patients, emphasizing the need for regular screening. Psychological disorders are more frequent in IBD populations compared to controls. Sleep quality emerged as the most important predictor, meriting focus in future interventional research. Female patients and those who were requiring corticotherapy had a higher chance for a psychological disorder. Biological therapy was associated with lower levels of anxiety and stress but a higher probability of depressive symptoms.

Future studies should prioritize longitudinal designs, standardized assessment protocols for psychological distress, and deeper exploration of sleep quality to clarify affective patterns in IBD and guide targeted interventions to prevent the large burden of psychological disorders.

## Figures and Tables

**Table 1:** Demographic and baseline characteristics of the study population. Continuous variables (age, years of education, body mass index, and hours of sleep) are presented as the mean ± standard deviation (SD); BMI = body mass index.

		CD (n = 55)	UC (n = 90)	Control (n = 210)	*p*
Age		37.40 (33.99–40.81)	41.20 (37.80–44.6)	40.86 (38.82–42.9)	0.58
Female gender		33 (60%)	55 (61.1%)	130 (61.9%)	0.96
Civil status					**<0.01**
	Single	17 (30.9%)	21 (23.4%)	31 (14.7%)	
	Stable relationship	10 (18.2%)	15 (16.7%)	18 (8.6%)	
	Married	24 (43.6%)	45 (50%)	154 (73.3%)	
	Divorced	2 (3.6%)	6 (6.6%)	2 (1%)	
	Widowed	2 (3.6%)	3 (3.3%)	5 (2.4%)	
Urban living area		51 (92.7%)	75 (83.3%)	180 (85.7%)	0.26
Professional activity					0.44
	Employee	41 (74.5%)	55 (61.1%)	155 (69%)	
	Retired	6 (12.7%)	16 (17.8%)	40 (19%)	
	Unemployed	1 (1.8%)	5 (5.6%)	2 (1%)	
	Student, trainee	6 (10.9%)	12 (13.3%)	18 (8.6%)	
Education (years)		13.91 (13.05–14.77)	15.42 (14.71–16.13)	15.51 (15.03–15.99)	**<0.01**
BMI (kg/m^2^)		24.72 (23.64–25.81)	24.12 (23.12–25.12)	25.77 (25.02–26.52)	0.097
Hours of sleep		6.79 (6.58–7)	6.70 (6.51–6.89)	6.73 (6.61–6.85)	0.97

**Table 2:** Clinical characteristics of patients with CD and UC (IBD = inflammatory bowel disease; 5ASA = 5amynosalycilates).

		CD (n = 55)	UC (n = 90)	*p*
Time since IBD diagnosis				0.69
Less than 1 year		3 (5.4%)	9 (10%)	
1–5 years		24 (43.6%)	41 (45.5%)	
6–10 years		15 (27.2%)	19 (21.1%)	
Over 10 years		13 (23.6%)	21 (23.3%)	
Specific dietary regimen		10 (18.1%)	19 (21.1%)	0.66
IBD complications		29 (52.7%)	24 (26.6%)	**<0.01**
Surgical treatment of complications		13 (23.6%)	4 (4.4%)	**<0.01**
Cortico-dependence		10 (18.1%)	9 (10%)	0.15
Disease modifying therapy				0.43
5ASA		12 (21.6%)	43 (47.7%)	
Biologic		32 (59%)	32 (35.4%)	
Immunosuppressor		4 (7.2%)	4 (4.4%)	
Homeopathic or probiotic		0 (0%)	4 (4.4%)	
None		7 (12.7%)	7 (7.7%)	

**Table 3:** Distribution of risk factors across the CD, UC, and control groups.

		CD (n = 55)	UC (n = 90)	Control (n = 210)	*p*
Smoking status					0.18
	Yes	17 (30.9%)	21 (23.3%)	57 (27.1%)	
	No	29 (52.7%)	62 (68.8%)	140 (66.6%)	
	Former smoker	8 (14.5)	7 (7.7%)	13 (6.1%)	
Standard of living					**<0.01**
	Difficult	12 (21.8%)	13 (14.4%)	6 (2.8%)	
	Decent	18 (32.7%)	31 (34.4%)	83 (39.5%)	
	Good	24 (43.6%)	43 (47.7%)	100 (47.6%)	
	Very good	1 (1.8%)	3 (3.3%)	21 (10%)	
Reported rest (Yes)		30 (54.5%)	52 (57.7%)	141 (67.1%)	0.11
Physical activity (Yes)		19 (34.5%)	48 (53.3%)	111 (52.8%)	**0.04**
Alcohol consumption (Yes)		21 (38.2%)	39 (56.7%)	142 (32.4%)	**<0.01**

**Table 4:** Stress, anxiety and depression scores among the three groups. (a) No significant differences between two groups. (b) No significant differences between two groups.

		CD (n = 55)	UC (n = 90)	Control (n = 210)	*p*
Stress level					**<0.01**
	Normal	31 (56.4%) (a)	47 (52.2%) (a)	164 (78.1%) (b)	
	Mild	8 (14.5%) (a)	11 (12.2%) (a)	19 (9%) (a)	
	Moderate	7 (12.7%) (a)	8 (8.9%) (a)	17 (8.1%) (a)	
	Severe	3 (5.5%) (a) (b)	11 (12.2%) (b)	4 (1.9%) (a)	
	Very severe	6 (10.9%) (a)	13 (14.4%) (a)	6 (2.9%) (b)	
Anxiety level					**0.016**
	Normal	24 (43.6%) (a)	35 (38.9%) (a)	104 (49.5%) (a)	
	Mild	5 (9.1%) (a)	11 (12.2%) (a)	46 (21.9%) (a)	
	Moderate	9 (16.4%) (a)	16 (17.8%) (a)	27 (12.9%) (a)	
	Severe	6 (10.9%) (a)	8 (8.9%) (a)	13 (6.2%) (a)	
	Very severe	11 (20%) (a) (b)	20 (22.2%) (a)	20 (9.5%) (a)	
Depression level					**<0.01**
	Normal	31 (56.4%) (a) (b)	48 (53.3%) (b)	148 (70.5%) (a)	
	Mild	6 (10.9%) (a)	5 (5.6%) (a)	23 (11%) (a)	
	Moderate	6 (10.9%) (a)	14 (15.6%) (a)	25 (11.9%) (a)	
	Severe	4 (7.3%) (a)	7 (7.8%) (a)	5 (2.4%) (a)	
	Very severe	8 (14.5%) (a)	16 (17.8%) (a)	9 (4.3%) (b)	

**Table 5:** Health-related quality-of-life assessment across the CD, UC, and control groups. (a) No significant difference between groups. (b) No significant difference between groups.

	CD (n = 55)	UC (n = 90)	Control (n = 210)	*p*
General Health Status (mean, mean ± SD)	76.16 (70.49–81.84) (a)	79.72 (76.22–83.22) (a)	90.31 (88.18–92.44) (b)	**<0.01**
Anxiety and Depression				**<0.01**
I am not anxious or depressed	28 (50.9%) (a)	41 (45.6%) (a)	141 (81.4%) (b)	
I am slightly anxious or depressed	14 (25.5%) (a)	25 (27.8%) (a)	22 (10.5%) (b)	
I am moderately anxious or depressed	8 (14.5%) (a) (b)	14 (15.6%) (b)	11 (5.2%) (a)	
I am severely anxious or depressed	4 (7.3%) (a)	6 (6.7%) (a)	6 (2.9%) (a)	
I am extremely anxious or depressed	1 (1.8%) (a) (b)	4 (4.4%) (b)	0 (0%) (a)	
Usual Activities				**<0.01**
I have no problems doing my usual activities	31 (56.4%) (a)	60 (66.7%) (a)	177 (84.3%) (b)	
I have slight problems doing my usual activities	18 (32.7%) (a)	21 (23.7%) (a), (b)	27 (12.9%) (b)	
I have moderate problems doing my usual activities	6 (10.9%) (a)	8 (8.9%) (a)	4 (1.9%) (b)	
I have severe problems doing my usual activities	0 (0%) (a)	1 (1.1%) (a)	1 (0.5%) (a)	
I am unable to do my usual activities	0 (0%) (a)	0 (0%) (a)	1 (0.5%) (a)	
Self-Care				**0.02**
I have no problems washing or dressing myself	41 (74.5%) (a)	70 (77.8%) (a)	188 (89.5%) (b)	
I have slight problems washing or dressing myself	11 (20%) (a)	16 (17.8%) (a)	20 (9.5%) (a)	
I have moderate problems washing or dressing myself	1 (1.8%) (a)	1 (1.1%) (a)	2 (1%) (a)	
I have severe problems washing or dressing myself	2 (3.6%) (a)	3 (3.3%) (a)	0 (0%) (b)	
Pain and Discomfort				**<0.01**
I have no pain or discomfort	29 (52.7%) (a)	43 (47.8%) (a)	165 (78.6%) (b)	
I have slight pain or discomfort	13 (23.6%) (a),(b)	31 (34.4%) (b)	34 (16.2%) (a)	
I have moderate pain or discomfort	11 (20%) (a)	15 (16.7%) (a)	7 (3.3%) (b)	
I have severe pain or discomfort	2 (3.6%) (a)	1 (1.1%) (a)	4 (1.9%) (a)	
Mobility				**0.015**
I have no problems walking	43 (78.2%) (a)	74 (82.2%) (a), (b)	190 (90.5%) (b)	
I have slight problems walking	9 (16.4%) (a)	12 (13.3%) (a)	16 (7.6%) (a)	
I have moderate problems walking	3 (5.5%) (a)	4 (4.4%) (a)	3 (1.4%) (a)	
I have severe problems walking	0 (0%) (a)	0 (0%) (a)	1 (0.5%) (a)	

**Table 6:** Multiple linear regression for predicting the square root of stress scores, adjusted for multiple covariates (CD = Crohn’s disease; BMI = body mass index; UC = ulcerative colitis).

Variable	B-	CI 95%	*p*
Stress score	2.841	(1.14, 4.54)	**<0.01**
UC vs. control	1.040	(0.59, 1.49)	**<0.01**
CD vs. control	0.725	(0.14, 1.31)	**0.015**
Female sex	0.718	(0.39, 1.05)	**<0.01**
Corticosteroid treatment	0.438	(−0.28, 1.16)	**0.023**
Smoking	0.329	(−0.01, 0.67)	0.06
Standard of living	0.253	(−0.08, 0.58)	**0.013**
Physical activity	0.237	(−0.09, 0.56)	0.15
Alcohol consumption	0.129	(−0.21, 0.47)	0.45
Reported hours of sleep	0.107	(−0.07, 0.28)	0.24
BMI	0.005	(−0.03, 0.04)	0.75
Relationship status	−0.003	(−0.38, 0.37)	0.98
Age	−0.016	(−0.027, −0.005)	**<0.01**
Education (years)	−0.044	(−0.09, 0.01)	0.08
Urban residence	−0.063	(−0.54, 0.41)	0.79
Special diet	−0.149	(−0.75, 0.45)	0.62
Biologic treatment	−0.408	(−0.91, 0.10)	0.11
Self-reported rest	−0.509	(−0.84, −0.18)	**<0.01**

**Table 7:** Multiple linear regression for predicting the square root of anxiety scores, adjusted for multiple covariates (CD = Crohn’s disease; BMI = body mass index; UC = ulcerative colitis).

Variable	B-	CI 95%	*p*
Anxiety score	2.521	(0.736–4.306)	**<0.01**
UC vs. control	0.686	(0.22–1.15)	**<0.01**
CD vs. control	0.527	(−0.09–1.14)	0.09
Female sex	0.527	(0.19–0.86)	**<0.01**
Smoking	0.509	(0.15–0.87)	**<0.01**
Corticosteroid treatment	0.396	(−0.36–1.16)	0.3
Alcohol consumption	0.177	(−0.18–0.54)	0.32
Special diet	0.158	(−0.48–0.79)	0.62
Reported hours of sleep	0.128	(−0.06–0.32)	0.18
Physical activity	0.067	(−0.28–0.41)	0.7
Relationship status	0.048	(−0.35–0.45)	0.81
Standard of living	0.043	(−0.31–0.40)	0.8
Age	−0.006	(−0.02–0.01)	0.34
BMI	−0.009	(−0.04–0.02)	0.6
Education (years)	−0.046	(−0.10–0.01)	0.08
Urban residence	−0.076	(−0.57–0.42)	0.76
Biologic treatment	−0.472	(−1.00–0.06)	0.08
Self-reported rest	−0.629	(−0.98–−0.28)	**<0.01**

**Table 8:** Multiple linear regression for predicting the square root of depression scores, adjusted for multiple covariates (CD = Crohn’s disease; BMI = body mass index; UC = ulcerative colitis).

Variable	B-	CI 95%	*p*
Depression score	2.764	(0.740–4.788)	**<0.01**
UC vs. control	0.812	(−0.284–1.340)	**<0.01**
Female sex	0.642	(0.256–1.027)	**<0.01**
Corticosteroid treatment	0.523	(−0.331–1.376)	0.22
Biologic treatment	0.493	(−0.109–1.096)	0.1
Physical activity	0.350	(−0.042–0.742)	0.08
CD vs. control	0.321	(−0.374–1.016)	0.36
Smoking	0.158	(−0.253–0.569)	0.44
Reported hours of sleep	0.112	(−0.102–0.326)	0.3
Relationship status	0.051	(−0.394–0.496)	0.82
Urban residence	0.019	(−0.544–0.583)	0.94
Standard of living	0.017	(−0.378–0.413)	0.93
BMI	−0.005	(−0.042–0.032)	0.8
Age	−0.016	(−0.029–−0.002)	**0.021**
Alcohol consumption	−0.035	(−0.439–0.369)	0.86
Education (years)	−0.056	(−0.116–0.004)	0.06
Special diet	−0.480	(−1.202–0.242)	0.19
Self-reported rest	−0.817	(−1.217–−0.417)	**<0.01**

## Data Availability

The original contributions presented in this study are included in the article. Further inquiries can be directed to the corresponding author.

## Abbreviations

IBD = inflammatory bowel disease; CD = Crohn’s disease; UC = ulcerative colitis; BMI = body mass index; VIF = variance inflation factor; IQR = interquartile range; 5ASA = 5 aminosalycilates; RR = relative risk; CI = confidence interval; GBA = gut–brain axis; SD = standard deviation.
